# Quantitative and Qualitative Assessments of Retinal Structure with Variable A-Scan Rate Spectralis OCT: Insights into IPL Multilaminarity

**DOI:** 10.3390/jcm12072637

**Published:** 2023-04-01

**Authors:** Marco Lupidi, Lorenzo Mangoni, Chiara Centini, Gregorio Pompucci, Luca Lanzafame, Luca Danieli, Daniela Fruttini, Enrico Peiretti, Jay Chhablani, Cesare Mariotti

**Affiliations:** 1 Eye Clinic, Department of Experimental and Clinical Medicine, Polytechnic University of Marche, 60126 Ancona, Italy; 2 Fondazione per la Macula Onlus, Di.N.O.G.Mi., University Eye Clinic, 16132 Genova, Italy; 3 IRCCS—Fondazione Bietti, 00198 Rome, Italy; 4 Department of Medicine and Surgery, University of Perugia, S. Maria della Misericordia Hospital, 06123 Perugia, Italy; 5 Eye Clinic, Department of Surgical Sciences, University of Cagliari, 09124 Cagliari, Italy; 6 Department of Ophthalmology, University of Pittsburgh School of Medicine, Pittsburgh, PA 15213, USA

**Keywords:** OCT, spectral domain OCT, retinal thickness, A-scan rate, inner plexiform layer, IPL

## Abstract

The aim of this study was to evaluate the qualitative and quantitative differences between 20 and 85 kHz A-scan rate optical coherence tomography (OCT) images acquired by spectral domain OCT. The study included 60 healthy subjects analyzed with horizontal linear scans with a variable A-scan rate (SHIFT technology, Heidelberg Engineering, Heidelberg, Germany). The retinal thickness measurement of each retinal layer was performed in three different positions (subfoveal, nasal, and temporal). The qualitative assessment was performed by two independent observers who rated every image with a score ranging from 1 (“sufficient”) to 3 (“excellent”) on the basis of three parameters: visualization of the vitreo-retinal interface, characterization of the retinal layers, and visualization of the sclero-choroidal interface. No statistically significant differences in terms of retinal layer thickness between the two A-scan rate scans were observed (*p* > 0.05). The coefficient of variation of the retinal thickness values was lower in the 20 kHz group (25.8% versus 30.1% with the 85 kHz). The 20 kHz images showed a higher quality index for both observers. An inner plexiform layer (IPL) multilaminarity was detected in 78.3% of patients from the 20 kHz group and in 40% of patients from the 85 kHz group (*p* < 0.05).

## 1. Introduction

Optical coherence tomography (OCT) is the gold standard for the diagnosis and management of an increasing variety of retinal disorders [1].

The introduction of the spectral-domain technology has allowed further progress, such as an increased acquisition rate of the image, higher tissue resolution by enhancing the sensitivity, and a significant reduction of eye motion artifacts caused during the examination [2].

Out of all the innovations that this field of technology is going through, one of the most relevant might be the SHIFT technology (Heidelberg Engineering, Heidelberg, Germany) [3]; this approach empowers the clinician to decide, case by case, the A-scan rate of an OCT [4,5]. A standard A-scan rate in Spectralis OCT (Heidelberg Engineering, Heidelberg, Germany) is set at 85 kHz, but given the possibility to “slow down” the A-scan rate to 20 kHz, we can elevate the sensitivity and improve, therefore, the image quality.

The SHIFT technology allows OCT-based patient assessment customization enhancing the detail of every retinal layer.

One of the retinal layers with the higher structural complexity is the inner plexiform layer (IPL). IPL contains the synapses between amacrine cells, bipolar cells, and retinal ganglion cells. The IPL has a double organization: bisublaminar (sublamina A and B, S-A, and S-B) based on bipolar cell axon terminations and pentalaminar (S1–S5) based on amacrine cell dendritic stratification [6,7]. The bisublaminar division is representative of ON (sublamina B) and OFF (sublamina A) vision pathways, which respectively depolarized and hyperpolarized in response to light stimulation [8,9,10]. The pentalaminar stratification can be observed in vivo with experimental OCT devices, but currently few reports have described it [11,12,13,14].

Potentially, the OCT imaging of the IPL could be a novel diagnostic biomarker to detect localized scattering changes that may be associated with pathway-dependent disease processes [12].

The aim of this study was to evaluate the presence of any qualitative or quantitative differences in the acquisition of foveal-passing OCT lines with an A-scan rate of 85 kHz and 20 kHz in healthy patients. The qualitative assessment included the presence or absence of a detectable multilaminarity of the IPL.

## 2. Materials and Methods

### 2.1. Patients’ Selection

This is a prospective study conducted at the Eye Clinic, Polytechnic University of Marche, Ancona, Italy. Sixty eyes of sixty healthy subjects were included in the study, between May and June 2022.

The patients had to be ≥18 years old and without any known ocular pathology to be included in the study. Their refractive status had to range between +4D and −6D.

Patients who underwent any prior ocular surgery or who suffered from any ocular condition (e.g., glaucoma) were excluded from the study, such as patients suffering from diabetes, systemic hypertension, or any other disease that could affect somehow the eye. Patients who took any drugs that potentially could cause ocular adverse effects were not included in the study as well.

The study adhered to the tenets of the Declaration of Helsinki. Written informed consent was obtained from all the subjects included in the study.

### 2.2. Ophthalmic Evaluations

After collecting a quick medical history to investigate if the patients met the inclusion and exclusion criteria, they underwent different ophthalmic examinations as the refractive status, a complete ophthalmic assessment, and optical biometry (AL-Scan, Nidek Medical srl, Japan). Finally, a spectral domain SD-OCT exam was performed using Spectralis OCT.

### 2.3. OCT Settings

The OCT horizontal foveal-centered linear scans were obtained using the automated image alignment eye-tracking software (Tru-Track^®^). The scan angle was set at 30° (9.4 mm), the images were captured in high resolution (HR) mode with 512 A-scans per 10° of field, and the ART mode (automated real-time averaging) was set on 100 frames. Using the SHIFT technology, the A-scan rate was set at 85 kHz for the first acquisitions and then switched to 20 kHz for the second ones. Only images whose quality was ≥30 dB were considered useful for the qualitative and quantitative assessment.

### 2.4. Quantitative Assessment

The OCT scans were then analyzed by the Heyex Software Version 6.7. (Heidelberg Engineering, Heidelberg, Germany).

**Figure 1:**
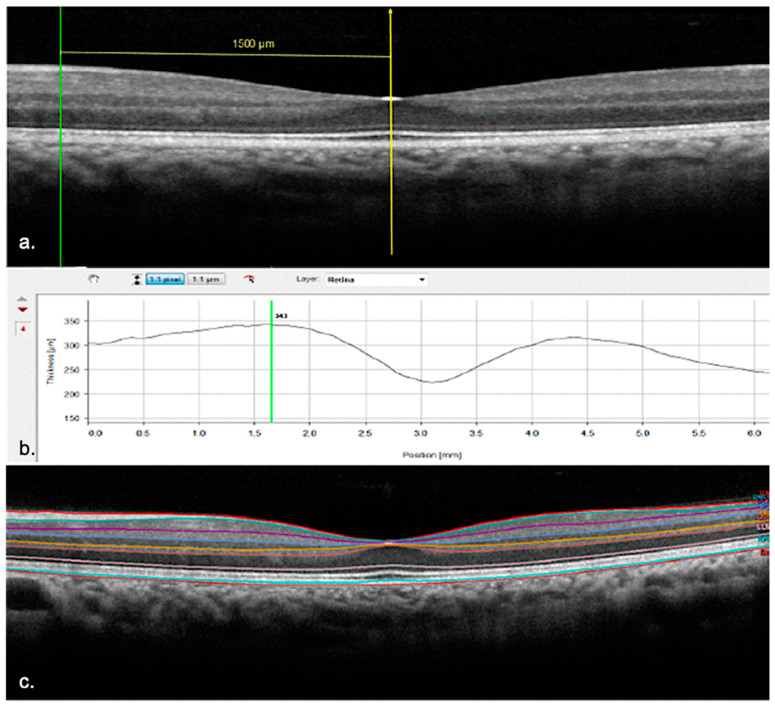
(**a**) Positioning of the caliper measuring 1500 microns nasally from the fovea. (**b**) Thickness measurement performed by Heyex Segmentation Software. (**c**) Segmentation of the individual retinal layers with colored lines imposed on the interfaces.

The measurements of every single retinal layer thickness were collected in three positions: subfoveally, 1500 microns nasally, and temporally from the foveal center. The extrafoveal locations were measured with the in-built “Caliper Tool” (Figure 1a). The thickness values of the following layers were recorded: retinal nerve fibers layer (RNFL), ganglion cells layer (GCL), IPL, inner nuclear layer (INL), outer plexiform layer (OPL), outer nuclear layer (ONL), and outer retina layers (ORL—included in between the external limiting membrane and the Bruch’s membrane) (Figure 1b,c).

### 2.5. Qualitative Assessment

**Figure 2:**
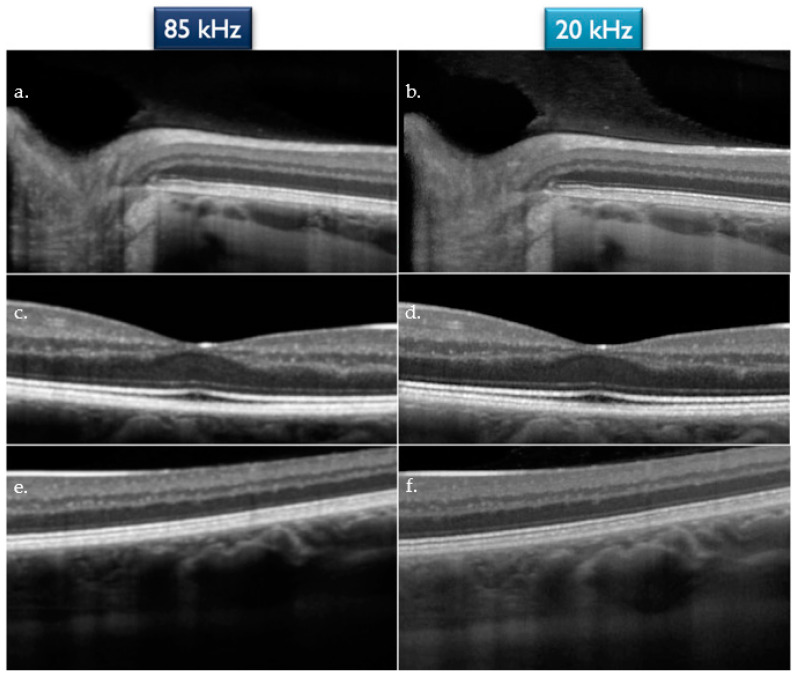
(**a**,**b**) Visualization of the vitreo-retinal interface for the 85 kHz and 20 kHz OCT, respectively. (**c**,**d**) Characterization of the retinal layers for the 85 kHz and 20 kHz OCT, respectively; (**e**,**f**) Visualization of the sclero-choroidal interface for the 85 kHz and 20 kHz OCT, respectively.

The OCT scans were then evaluated by two independent observers, who rated every single image with a score ranging from 1 to 3, where 1 was for “Sufficient”, 2 for “Good”, and 3 for “Excellent”. The parameters that have been judged in order to assess the quality were: visualization of the vitreo-retinal interface, characterization of the retinal layers, and visualization of the sclero-choroidal interface. If these three features were all sharply and clearly visualized by the observer, the grading was “Excellent”; if only two of them, the grading was “Good” and finally if only one of them, the grading was “Sufficient” (Figure 2).

The observers were masked to the A-scan rate of each image. Each observer graded every image three times. No statistically significant differences were found between the intra-observer evaluations (*p* > 0.05). The median value was assumed as the final grading. 

The values were then coupled into two categories: the values “1 and 2” are coupled as the “≤2” category whereas the values “3” stood alone as the category “3” for statistical purposes.

Moreover, the observers were asked if the IPL multilaminarity could be detectable in the images. The answers were recorded in a binary fashion: “yes” or “no”.

### 2.6. Statistical Analysis

Descriptive statistics (mean, standard deviation, coefficient of variation) have been analyzed for the whole parameters.

For quantitative data, the Kolmogorov–Smirnov test was used to establish whether the data were distributed normally. Based on the results obtained, the Wilcoxon–Mann–Whitney non-parametric test for repeated measures or the *t*-test for paired data was used to compare the two groups.

Spearman’s R index was used to calculate the correlation between the quantitative and qualitative variables of the two groups and with the refractive characteristics. The intraclass correlation coefficient (ICC) was used to assess agreement between the observers for the qualitative evaluation. Interpretation of ICC was based on the following guideline: below 0.50, poor; 0.50–0.75, moderate; 0.75–0.90, good; >0.90, excellent [15].

Bland–Altman plots illustrate differences in measures between Spectralis OCT 20 kHz and 85 kHz (*y*-axis) against the average of measures from the same techniques (*x*-axis). Bland–Altman limits of agreement (LOA) demonstrate where 95% of the data points should lie within ±1.96 standard deviations of the mean difference. The mean difference is the average difference between the methods assessed.

Fisher’s exact test has been applied to the qualitative data and for the assessment of the multilaminarity of the IPL. The significance level was set at *p* < 0.05. The statistical analysis was performed with the software IBM SPSS V. 25.0.0.

## 3. Results

Sixty eyes of 60 patients (28 were female, 46.7%) were enrolled in this study. The mean age was 37.6 years (SD 11.8).

The mean refractive status was −1.18 D (±2.14 D) and the mean axial length was 24.18 mm (±0.97 mm). The mean value of K1 was 42.98 D (±1.79 D) and the mean value of K2 was 43.69 D (±1.81 D).

### 3.1. Quantitative Assessment

The thickness values of each retinal layer obtained by OCT scans with an A-scan rate of 20 kHz and 85 kHz are reported in Table 1 divided into foveal, nasal, and temporal measurements.

No statistically significant differences in terms of retinal layer thickness were observed (*p* > 0.05).

The coefficients of variation (COV) were calculated for each layer thickness measured on the two different A-scan rate scans (20 kHz and 85 kHz). The 20 kHz OCT showed a COV inferior to the 85 kHz OCT’s one in 17 out of 24 layers. The overall COV was inferior in the 20 kHz group (25.8%) compared to the 85 kHz group (30.1%). The values are resumed in Table 2.

Bland–Altman plots illustrate the differences in measures between Spectralis OCT 20 kHz and 85 kHz (*y*-axis) against the average of measures from the same techniques (*x*-axis), showing the agreement between the two methods. Bland–Altman limits of agreement (LOA) demonstrate where 95% of the data points should lie within ±1.96 standard deviations of the mean difference. The mean difference is the average difference between the methods assessed.

**Figure 3:**
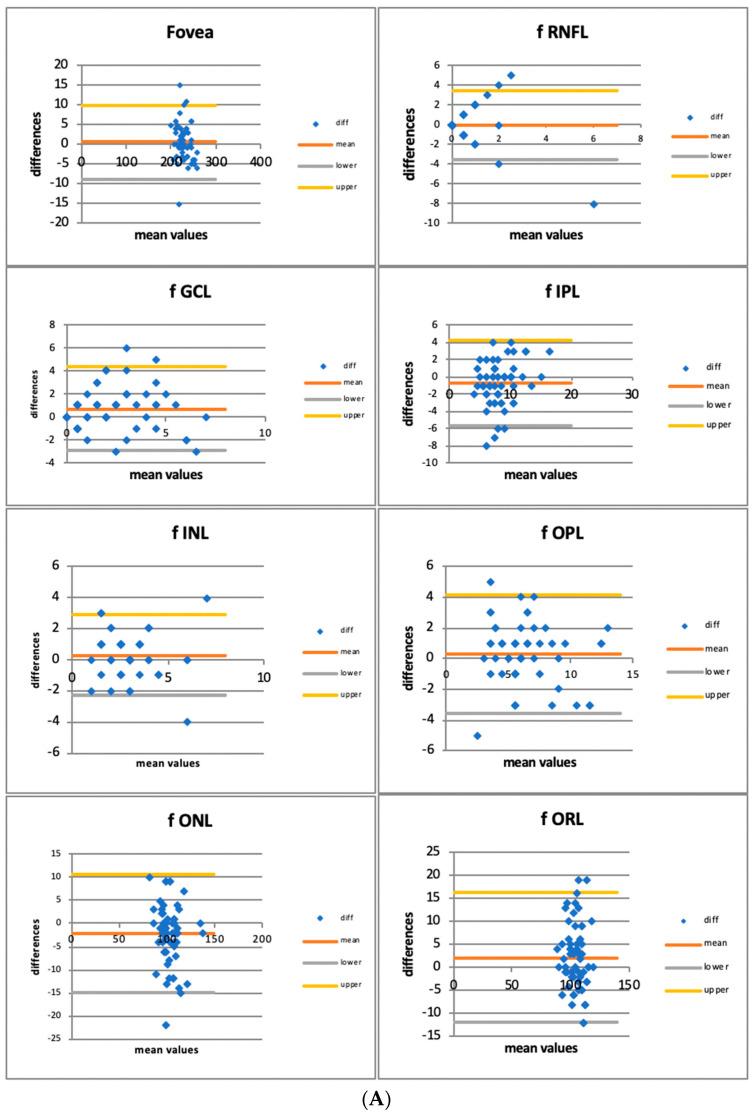

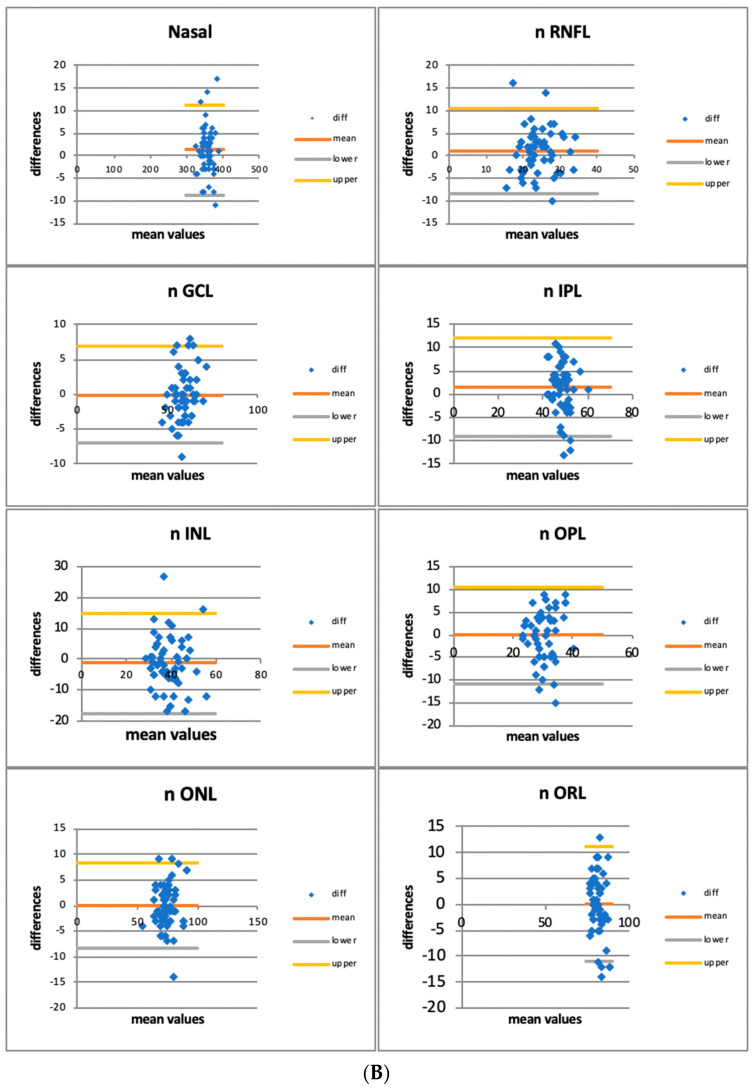

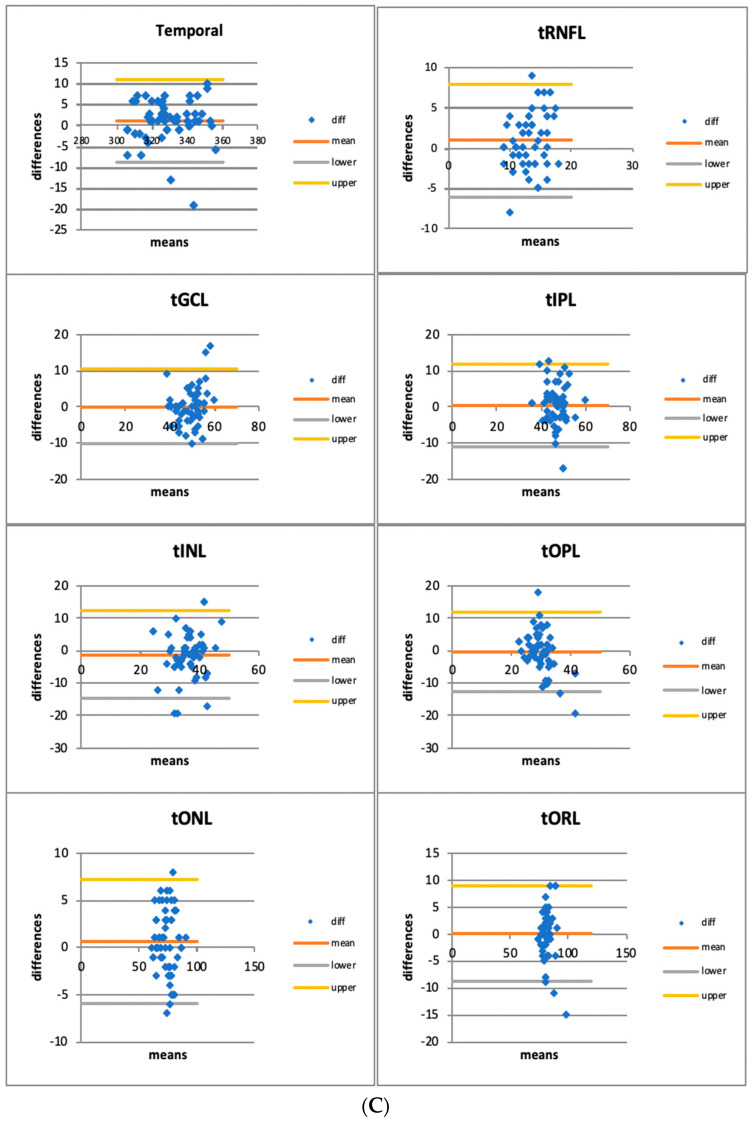
(**A**) Bland–Altman Plots for retinal layers thickness in the foveal area (f = foveal; RNFL: retinal nerve fiber layer; GCL: ganglion cell layer; IPL: inner plexiform layer; INL: inner nuclear layer; OPL: outer plexiform layer; ONL: outer nuclear layer; ORL: outer retinal layer). (**B**) Bland–Altman Plots for retinal layers thickness in the nasal area (n = nasal; RNFL: retinal nerve fiber layer; GCL: ganglion cell layer; IPL: inner plexiform layer; INL: inner nuclear layer; OPL: outer plexiform layer; ONL: outer nuclear layer; ORL: outer retinal layer). (**C**) Bland–Altman Plots for retinal layers thickness in the temporal area (t = temporal; RNFL: retinal nerve fiber layer; GCL: ganglion cell layer; IPL: inner plexiform layer; INL: inner nuclear layer; OPL: outer plexiform layer; ONL: outer nuclear layer; ORL: outer retinal layer).

The plots are resumed in the following graphs (Figure 3A–C).

### 3.2. Qualitative Assessment

The mean value for the first observer grading was 2.67 (SD 0.572) for the 20 kHz images and 2.28 (SD 0.691) for the 85 kHz images. The difference was statistically significant (*p* = 0.007).

The mean value for the second observer grading was 2.58 (SD 0.591) for the 20 kHz images and 2.33 (SD 0.705) for the 85 kHz images. The difference was statistically significant (*p* = 0.043).

No statistically significant differences between the two operators were reported in the grading of the 20 kHz scans (*p* = 0.133) or the 85 kHz scans (*p* = 0.410).

**Figure 4:**
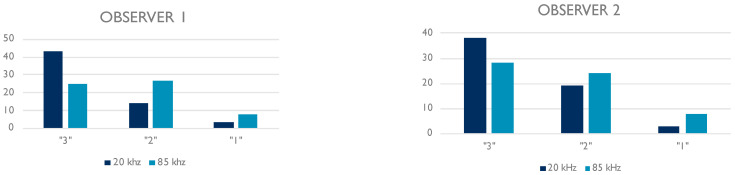
Histograms showing the qualitative assessments provided by each observer to the 20 kHz and 85 kHz OCT images.

The quality data are resumed in Table 3 and in Figure 4.

The qualitative data assessed by the two observers showed a good positive correlation rate by the intra-observer Spearman’s correlation test (R):

- 20 kHz Observer 1–Observer 2: R = 0.735 (*p* < 0.001)
- 85 kHz Observer 1–Observer 2: R = 0.777 (*p* < 0.001).

Furthermore, the intraclass correlation coefficient (ICC) showed a good agreement between the observers both for the 20 kHz images (ICC = 0.82) and for the 85 kHz images (ICC = 0.83).

The data were then coupled into two categories: the values “1 and 2” are paired as “≤ 2” category whereas the value “3” stood alone as the category “3”. That allowed us to apply the Fisher’s exact test to these variables which showed a statistically significant difference for both observers (observer 1: *p* = 0.0079; observer 2: *p* = 0.0031)

**Figure 5:**
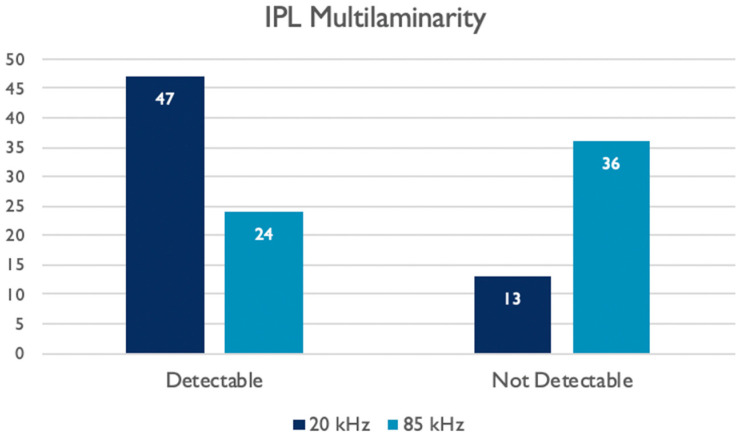
Histograms showing the number of images where the IPL multilaminarity was detectable.

The multilaminarity of IPL was detectable in 47 patients in the 20 kHz images (78.3%) and in 24 patients in the 85 kHz images (40%) (Figure 5). The Fisher’s exact test showed a statistically significant difference between the two imaging modalities (*p* = 0.0024). Data are reported in Table 4.

### 3.3. Correlation Analysis

The correlation analysis was performed using Spearman’s correlation test.

The correlations between the same retinal layer thicknesses measured by the two A-scan rates OCT are resumed in Table 5. Of the 24 layers analyzed, 20 showed a statistically significant correlation with Spearman’s R index and only four did not (foveal RNFL, nasal IPL, nasal ORL, and temporal OPL).

The correlation analysis performed between the qualitative assessments provided by each observer to the images at 20 kHz and 85 kHz showed a positive correlation with the multilaminarity detection. The results are resumed in Table 6.

## 4. Discussion

The quantitative results of this study revealed that there are no significant differences between the two A-scan rates OCT in the thickness measurements of the retinal layers. The 85 kHz A-scan rate OCT is a reliable and reproducible diagnostic tool in retinal imaging [16,17,18]. The 20 kHz A-scan rate OCT demonstrated a comparable reproducibility in terms of the accuracy of retinal layer segmentation. Indeed, the correlation analysis showed a strong correlation in thickness measurement of the same layer with the two different A-scan rate patterns in almost all retinal layers. The overall COV for the 20 kHz is 25.8% whereas for the 85 kHz is 30.1%. We can speculate that the sensitivity of the images scanned with the 20 kHz modality is higher than the 85 kHz one, thus leading to a more accurate value of thickness measurement through the analysis of a sharper image. Nevertheless, the limited difference allows us to consider the 85 kHz as a good balance between scanning speed and image quality for any quantitative assessment.

As it is known, the sensitivity of an OCT system describes the largest permissible signal attenuation within a sample that can still be distinguished from the noise. In practice, higher-sensitivity OCT systems are capable of providing higher-contrast images. As the sensitivity of an OCT system can be increased by increasing the integration time, there is usually a trade-off between the A-scan rate and sensitivity [19].

As integration time is the time period during which one A-scan is captured, it is inversely related to acquisition rate and there is a trade-off between sensitivity and acquisition speed. Increasing the A-scan rate by a factor of 2 leads to a 50% loss of integration time and therefore to a 50% loss of sensitivity, which translates to 3 dB in the conventional sensitivity units [20].

So decreasing the A-Scan Rate from 85 kHz to 20 kHz leads to an increase in the integration time, which conversely results in a signal-to-noise ratio (SNR) improvement, as shown in the following equation [21]. (*SNR_SD_*: signal-to-noise ratio of spectral domain OCT; *η*: conversion factor; *P_sample_*: sample arm power; *τ_i_*: integration time; *E_v_*: photon energy at an optical frequency) [21].

$${SRN}_{SD}^{\left(shot\right)}=\frac{\eta P_{sample}\tau_{i}}{E_{v}}$$

That is in accordance with the qualitative assessment ruled out in this study, in which the quality values resulted higher using the 20 kHz OCT than using the 85 kHz one (2.67 and 2.58 versus 2.28 and 2.33) with a 71.7% of “Excellent” (i.e., “3” value) labeled imagines with the first method.

Switching the A-scan rate to 20 kHz, with an increased integration time, leads to a slower speed of acquisition and a longer acquisition time [3]. The SHIFT technology allows switching the A-scan rate also to 125 kHz if a shorter acquisition time is needed, e.g., in patients with fixation problems. Indeed, on the other hand, the improved sensitivity achieved by 20 kHz imaging allows us to acquire clearer scans in challenging eyes with media opacities such as corneal edema or scars severe dry eye, cataracts, or dense floaters [3]. Furthermore, it allows also an enhanced definition of the retinal structure. 

In fact, in the 20 kHz images it was possible to detect a multilaminarity in the IPL layer in 78.3% of cases, against 40% with the 85 kHz. The difference was statistically significant as confirmed by the Fisher’s exact test (Table 4) and showed a positive correlation with the quality index assessed by the two observers (Table 6). Thus, it leads to speculation that when the quality and the sensibility of the scans improve and the signal-to-noise ratio increases, then the microscopical structure of the IPL could be more easily detectable.

The presence of this multilaminarity has been proved by several histological studies ex vivo by immunostaining of various cell types [22,23,24].

The IPL contains synapses between bipolar cells, amacrine cells, and the output ganglion cells. It is divided into ON (sublamina B) and OFF (sublamina A) bipolar cell axon terminations, which give rise to ON and OFF channels [8] that nominally respond to light increments and decrements, respectively. The IPL is often further divided into five strata of approximately equal thickness, with the two innermost strata corresponding to the ON pathway (sublamina B), the two outermost strata corresponding to the OFF pathway (sublamina A), and the middle stratum designated as either ON or as a watershed zone [7]. This pentalaminar scheme for describing the IPL has now become a de facto convention.

Although the function of the ON and OFF pathways can be individually assessed non-invasively by electroretinography or electroencephalography, there is no known in vivo methodology that can assess their anatomy. Perhaps the closest approach is OCT, a standard clinical imaging modality for in vivo high-resolution cross-sectional imaging of the human retina [25].

Conveniently, the laminar organization of the retina, with synaptic layers alternating with nuclear layers, leads to differences in reflectivity (backscattering) that form the basis for OCT image contrast [1]. However, although the IPL and OPL are well-visualized in OCT, the internal structure of these layers has received little attention, aside from a few scattered reports noting the presence of IPL stratification [11,12,14].

One possible reason is that the changes in reflectivity detectable in the sub-stratification of a synaptic layer are subtler than those that give rise to the contrast between a nuclear and a synaptic layer. Moreover, retinal stratification occurs on the micron scale, requiring depth resolution beyond the capabilities of most commercial NIR OCT systems to distinguish. In fact, all of these studies were performed using non-commercial broadband OCT systems [11,12,14].

**Figure 6:**
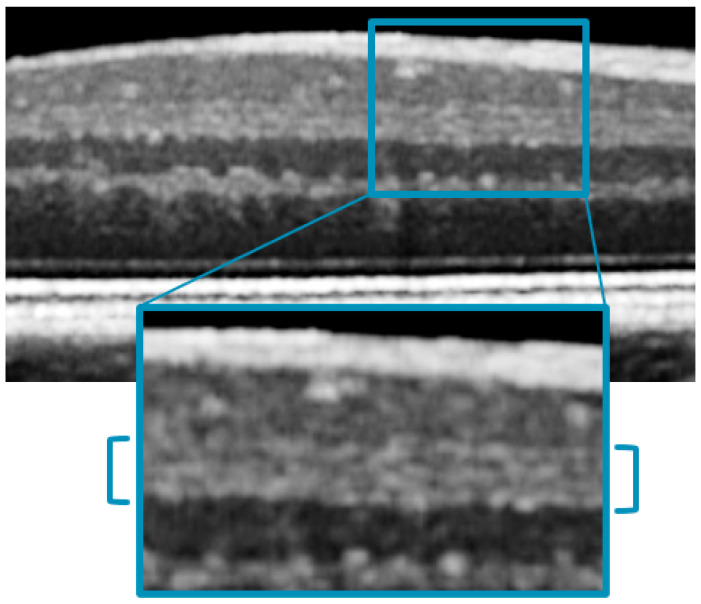
Magnification of a 20 kHz OCT scan, showing the visible pentalaminar pattern of the inner plexiform layer (IPL).

As described by Zhang et al. [14], using a prototype visible light OCT system developed at UC Davis, the image-averaged IPL profile analysis showed the characteristic pentalaminar pattern with three hyper-reflective strata (S1, S3, and S5) separated by two hypo-reflective strata (S2 and S4). This same pattern was visible, even if with lower contrast, with the 20 kHz Spectralis OCT that we used in our study (Figure 6).

## 5. Conclusions

Imaging with 20 kHz Spectralis OCT resulted in an accurate and reliable tool for daily ophthalmological practice.

It has been shown that this could be more sensitive than the standard 85 kHz OCT and improves the image quality, especially in those eyes with media opacities, such as cataracts, corneal edema, or dense floaters [3].

We demonstrated that this approach can be beneficial also in healthy eyes, showing a substantial qualitative improvement leading to a more accurate microstructural assessment of retinal layers.

The SHIFT technology offers the flexibility to change the A-scan rate (20 kHz, 85 kHz, 125 kHz) in order to find the perfect balance between image quality and speed and to provide customized patient care.

Further studies are required to evaluate if the microstructural assessment of the retinal layers could be further enhanced, as an in vivo visualization of the tissue details comparable to a microscopic ex vivo one.

That could open the path to the discovery of new markers or predictive factors also in pathological eyes and could give an insight into the sublayer’s behaviors in different retinal diseases.

## Figures and Tables

**Table 1:** Thickness measurements (μm) of the retinal layers assessed in the subfoveal, nasal, and temporal retina. Mean value and standard deviation were recorded. The *p* values are reported, and *p* < 0.05 were assumed as statistically significant (RNFL: retinal nerve fiber layer; GCL: ganglion cell layer; IPL: inner plexiform layer; INL: inner nuclear layer; OPL: outer plexiform layer; ONL: outer nuclear layer; ORL: outer retinal layer).

		20 kHz		85 kHz		
FOVEAL		Mean (μm)	SD	Mean (μm)	SD	*p*
	TOTAL	228.55	13.576	227.92	14.563	0.310
	RNFL	0.52	1.066	0.55	1.466	0.883
	GCL	2.58	2.102	2.61	2.051	0.521
	IPL	7.87	2.100	8.62	2.525	0.056
	INL	3.07	1.364	2.77	1.430	0.086
	OPL	6.56	2.380	6.19	2.757	0.130
	ONL	102.00	10.025	102.12	11.073	0.154
	ORL	105.87	7.386	102.58	12.730	0.053
NASAL		Mean (μm)	SD	Mean (μm)	SD	*p*
	TOTAL	356.40	15.075	355.17	15.107	0.066
	RNFL	24.75	4.963	23.70	4.816	0.099
	GCL	58.78	5.693	58.85	4.772	0.884
	IPL	48.93	3.970	48.73	4.876	0.484
	INL	38.32	7.149	39.50	7.659	0.276
	OPL	29.95	4.931	30.05	4.597	0.889
	ONL	74.05	7.559	74.07	7.095	0.976
	ORL	81.50	3.606	81.40	4.851	0.892
TEMPORAL		Mean (μm)	SD	Mean (μm)	SD	*p*
	TOTAL	330.08	14.377	328.88	13.748	0.072
	RNFL	13.60	3.396	13.32	2.451	0.346
	GCL	49.23	6.264	49.17	5.156	0.923
	IPL	46.90	4.874	46.40	5.493	0.511
	INL	35.63	6.175	36.93	5.713	0.150
	OPL	29.73	3.672	30.25	6.078	0.526
	ONL	73.55	7.210	72.58	7.777	0121
	ORL	81.57	4.327	81.55	4.659	0.977

**Table 2:** Coefficients of variation (COV) for each layer measure with 20 kHz A-scan rate OCT and with 85 kHz A-scan rate OCT. The values labeled in grey are the lower values for each layer (F = foveal, N = nasal, T = temporal; RNFL: retinal nerve fiber layer; GCL: ganglion cell layer; IPL: inner plexiform layer; INL: inner nuclear layer; OPL: outer plexiform layer; ONL: outer nuclear layer; ORL: outer retinal layer).

	COV 20 kHz	COV 85 kHz		COV 20 kHz	COV 85 kHz
FOVEAL	5.9%	6.4%	N INL	18.7%	19.4%
F RNFL	206.2%	266.6%	N OPL	16.5%	15.3%
F GPL	81.4%	108.9%	N ONL	10.2%	9.6%
F IPL	29.3%	39.4%	N ORL	4.4%	6.0%
F INL	44.5%	51.7%	TEMPORAL	4.4%	4.2%
F OPL	36.3%	44.4%	T RNFL	25.0%	19.5%
F ONL	9.8%	10.6%	TGPL	12.7%	10.5%
F ORL	7.0%	12.4%	T IPL	10.4%	11.8%
NASAL	4.2%	4.3%	T INL	17.3%	15.5%
N RNFL	20.1%	20.3%	T OPL	12.4%	20.1%
N GPL	9.7%	8.1%	T ONL	9.8%	10.7%
N IPL	8.1%	10.3%	T ORL	5.3%	5.7%

**Table 3:** Quality assessment values provided by the two observers for the 20 kHz images and 85 kHz images.

**20 kHz**		
	**Observer 1**	**Observer 2**
Grade “3”	43 (71.7%)	38 (63.3%)
Grade “2”	14 (23.3%)	19 (31.7%)
Grade “1”	3 (5%)	3 (5%)
**85 kHz**		
	**Observer 1**	**Observer 2**
Grade “3”	25 (41.7%)	28 (46.7%)
Grade “2”	27 (45%)	24 (40%)
Grade “1”	8 (13.3%)	8 (13.3%)

**Table 4:** Contingency table showing the number of images in which the IPL multilaminarity was detectable for the two different A-scan rate acquisitions.

IPL Multilaminarity Detection		20 kHz Images		-
		Not Detectable	Detectable	TOT
85 kHz Images	Not Detectable	4	32	36 (60, 0%)
	Detectable	9	15	24 (40, 0%)
	TOT	13 (21, 7%)	47 (78, 3%)	60 (100%)

**Table 5:** Spearman’s correlation index (R) between the two matching retinal layers’ thickness measured with a 20 kHz and an 85 kHz A-scan rate in the foveal, nasal, and temporal areas. The bold values are statistically siginificative (*p* < 0.05) (RNFL: retinal nerve fiber layer; GCL: ganglion cell layer; IPL: inner plexiform layer; INL: inner nuclear layer; OPL: outer plexiform layer; ONL: outer nuclear layer; ORL: outer retinal layer).

		FOVEAL	NASAL	TEMPORAL
TOTAL THICKNESS 20 vs. 85 kHz	R	**0.938**	**0.935**	**0.946**
	*p*	**0.001**	**0.001**	**0.001**
RNFL 20 vs. 85 kHz	R	−0.132	**0.551**	**0.317**
	*p*	0.315	**0.001**	**0.014**
GCL 20 vs. 85 kHz	R	**0.570**	**0.738**	**0.606**
	*p*	**0.001**	**0.001**	**0.001**
IPL 20 vs. 85 kHz	R	**0.516**	0.224	**0.313**
	*p*	**0.001**	0.085	**0.015**
INL 20 vs. 85 kHz	R	**0.547**	**0.378**	**0.324**
	*p*	**0.001**	**0.003**	**0.012**
OPL 20 vs. 85 kHz	R	**0.609**	**0.329**	0.154
	*p*	**0.001**	**0.010**	0.240
ONL 20 vs. 85 kHz	R	**0.754**	**0.817**	**0.826**
	*p*	**0.001**	**0.001**	**0.001**
ORL 20 vs. 85 kHz	R	**0.509**	0.163	**0.477**
	*p*	**0.001**	0.214	**0.001**

**Table 6:** Spearman’s correlation index (R) between the qualitative assessment of each observer and the multilaminarity detection of the same observer both to the 20 kHz images and to the 85 kHz. The bold values are statistically siginificative (*p* < 0.05).

		Multilaminarity Detection
Observer 1 20 kHz	R	**0.847**
	*p*	**<0.001**
Observer 2 20 kHz	R	**0.720**
	*p*	**<0.001**
Observer 1 85 kHz	R	**0.735**
	*p*	**<0.001**
Observer 2 85 kHz	R	**0.771**
	*p*	**<0.001**

## Data Availability

Data are fully available on the base of a specific and motivated request to the authors.
